# Digital transformation in laboratory documentation: Insights from an eLabFTW case study and a survey-informed onboarding framework

**DOI:** 10.1371/journal.pone.0355558

**Published:** 2026-08-20

**Authors:** Christian Jäger, Magdalena Zagrodnick, Benjamin Schwede, Jan Christoph

**Affiliations:** 1 Research Group (Bio-)Medical Data Science, Medical Faculty, Martin Luther University Halle-Wittenberg (MLU), Halle (Saale), Germany; 2 Data Integration Center (DIC), University Medicine Halle, Halle (Saale), Germany; Philadelphia University, JORDAN

## Abstract

**Purpose:**

This study addresses the implementation of the electronic laboratory notebook (ELN) eLabFTW at the University Medicine Halle (UMH) and collaborating departments at Martin Luther University Halle-Wittenberg. It explores how ELNs can improve research documentation by increasing efficiency, data quality, and alignment with (FAIR) principles, while also navigating institutional and user-specific challenges.

**Methods:**

A structured, multi-phase approach was followed, including stakeholder engagement, an interdisciplinary pilot phase, and technical integration into institutional infrastructure. Evaluation was conducted via a comprehensive user survey covering documentation practices, system requirements, and acceptance factors. The survey yielded 55 fully analyzable responses (*n* = 55). Additionally, custom Shiny applications and practical checklists were developed to streamline implementation and onboarding. These tools are published via GitHub and accompany this work as reusable resources for other research institutions.

**Results:**

Most respondents (78%) reported extensive documentation experience, but frequently cited challenges such as time burden (67%), inefficient organization (63%), and difficulties retrieving information (63%). Traditional tools such as spreadsheets (87%) and paper notebooks (85%) dominate, but 90% emphasized user-friendliness as a key requirement for ELN adoption. Structured training (71%) and ongoing technical support (52%) were also deemed essential for success. During the 22-month institutional rollout period (October 2024 to July 2026), the platform grew to 237 registered users across 32 research teams, with 4,263 active experiments created by the end of the observation period.

**Conclusion:**

The observed adoption demonstrates that a structured, user-centered implementation strategy can support the integration of an ELN into routine research practice. In addition to empirical insights, this publication provides openly accessible tools including Shiny apps and checklists that facilitate ELN onboarding and can support efficient implementation strategies in other institutional contexts. An important extension of the implementation was the integration of customizable dashboards, developed using Apache Superset and integrated into the institutional Confluence environment. These enable the secondary use of structured documentation data for visualization, quality monitoring, and reporting, providing additional analytical capabilities beyond the primary documentation function of the ELN. ELNs can support transparent, collaborative, and sustainable research data management when combined with structured implementation and institutional support.

## Introduction

The documentation of research results is a fundamental element of scientific work and a prerequisite for quality assurance, traceability, and reproducibility. Traditionally, laboratory documentation has been paper-based, which presents persistent challenges such as time-consuming organization, limited collaboration, and restricted data security [1]. Electronic Laboratory Notebooks (ELNs) offer a modern solution that can improve efficiency, transparency, and data integrity [2–4].

A successful transition from paper to electronic documentation requires more than introducing a new tool. It involves a systematic implementation strategy that balances user-friendliness, technical reliability, and institutional support. According to the Technology Acceptance Model by Davis et al. [5], perceived usefulness and ease of use are key factors determining the acceptance of new technologies. Consequently, ELN implementations should be guided by user-centered principles that address practical challenges and researcher’s expectations. Enhancing both usability and perceived value can substantially increase acceptance and ensure sustainable integration into daily scientific workflows.

The FAIR principles – Findable, Accessible, Interoperable, and Reusable – provide a widely adopted framework for sustainable research data management [6]. Electronic laboratory notebooks do not inherently ensure FAIR compliance. However, ELNs can support FAIR-oriented documentation practices when combined with structured metadata concepts, standardized templates, controlled vocabularies, and interoperable data structures.

In this context, the implementation of eLabFTW [7] at University Medicine Halle was accompanied by a strong emphasis on structured documentation practices during onboarding and training activities. Rather than merely storing uploaded documents, the implementation strategy aimed to encourage the structured capture of experimental metadata, reusable parameters, and harmonized documentation fields to improve the findability, comparability, and long-term reusability of research-related information.

Cultural aspects of data management have been emphasized by Valdez et al. [8], who examined data sharing practices among 563 researchers from 64 countries. The authors describe the so-called Gollum effect, referring to excessive data ownership and territoriality that hinder collaboration. Nearly half of the respondents reported encountering such behaviors, often from senior researchers or competing groups, with early-career scientists being most affected. These behaviors lead to restricted collaboration, reduced transparency, and even withdrawal from academic research. The findings underline the necessity for digital infrastructures such as institutional ELNs that promote openness, accountability, and FAIR-compliant practices through appropriate policies and structural support.

Although the present study did not directly assess behavioral changes related to data ownership or territoriality, several survey findings indicated practical challenges related to collaboration and information sharing. In particular, the survey further identified high demand for improvements in data findability, data linking, access possibilities, and collaboration support. Structured ELN environments can help address such technical and organizational barriers by enabling searchable documentation structures, standardized metadata, reusable templates, and controlled access management. In this context, ELNs may support more transparent and collaborative research workflows even though broader cultural factors influencing data sharing behavior extend beyond technical infrastructure alone.

This study documents the introduction of the electronic laboratory notebook eLabFTW at the University Medicine Halle (UMH), which includes the University Hospital Halle (UKH) and the Medical Faculty of Martin Luther University Halle-Wittenberg (MLU). The implementation aimed to modernize documentation across a diverse research landscape, extending beyond university medicine to affiliated departments throughout the university. This study makes three main contributions.

First, it provides an empirical assessment of current documentation practices, challenges, and user requirements in a heterogeneous research environment based on a structured user survey.

Second, it introduces a survey-informed framework for the systematic implementation and onboarding of electronic laboratory notebooks (ELNs), explicitly linking user needs to technical and organizational measures.

Third, it presents practical implementation resources, including R Shiny applications, structured onboarding materials, and Apache Superset-based dashboards that support ELN adoption and enable the visualization of structured documentation data in institutional settings. By combining empirical analysis, implementation strategy, and practical support tools, this work offers a reproducible and user-centered approach for institutions seeking to establish sustainable and FAIR-aligned digital research infrastructures.

## Milestones of implementation

### Decision-making process

The decision to implement an ELN at the Medical Faculty of Martin Luther University Halle-Wittenberg was reached after extensive discussions among different stakeholders and the Faculty Council. The main criteria included user-friendliness, data security, and adaptability to local research needs. After evaluating several options, eLabFTW was selected because it best fulfilled the institutional requirements regarding self-hosted deployment, long-term sustainability, interoperability, and integration into the existing infrastructure. Although self-hosted operation requires dedicated institutional resources for maintenance and support, the open-source model offers long-term flexibility, institutional control over system development, and independence from vendor-specific licensing models.

The selection of eLabFTW was based on a structured evaluation process that considered not only user expectations but also institutional, technical, organizational, and long-term sustainability requirements. Candidate systems were identified and compared using publicly available decision-support resources including the ELN Finder platform [9] and the PUBLISSO ELN-Filter [10]. Among the evaluated systems, openBIS and eLabFTW emerged as the most suitable candidates for further assessment.

While survey participants assigned comparatively low importance to licensing models, institutional decision-making required additional consideration of long-term scalability, sustainability, operational independence, and the implications of subscription-based software models for a large academic user base. Similar considerations have previously been described for other institutional ELN implementations [2].

For the heterogeneous and publicly funded research environment at University Medicine Halle, particular emphasis was placed on long-term sustainability, institutional control over research data, self-hosted deployment, interoperability, and integration into existing authentication infrastructures. In addition, the existing academic user community surrounding eLabFTW was considered advantageous for knowledge exchange, shared best practices, and long-term maintainability.

Taken together, these considerations led to the selection of eLabFTW as a flexible and sustainable platform capable of supporting institution-specific onboarding workflows, metadata standardization strategies, and FAIR-oriented documentation practices.

The adoption of the FAIR principles served as a guiding concept during this process. The commitment to make research data findable, accessible, interoperable, and reusable reflects the faculty’s strategic focus on transparent and reproducible research practices. These principles have been recognized internationally as a foundation for enabling interoperability, collaboration, and long-term data stewardship.

### Pilot phase

Before large-scale implementation, an eight-month pilot phase was conducted to assess the feasibility, usability, and adaptability of eLabFTW under real laboratory conditions. Interdisciplinary research groups from clinical, biomedical, and pharmaceutical research environments participated in the pilot phase, representing heterogeneous laboratory workflows and documentation practices. This phase was essential for identifying technical and organizational challenges, refining workflows, and validating system performance [11].

The evaluation combined iterative user feedback obtained through workshops, bilateral discussions, committee exchanges, training events, and surveys with technical assessments and practical implementation experience.

Feedback focused particularly on usability, workflow integration, metadata handling, onboarding requirements, access management, and compatibility with existing documentation routines. It included a comparative review of alternative ELN systems (eLabFTW and openBIS [12]), a criteria-based selection process, and a systematic evaluation of usability, workflow integration, and IT compatibility. The results emphasized the importance of flexible user and rights management, seamless integration into existing infrastructures, and accessible user support mechanisms.

Training and onboarding emerged as critical success factors, particularly for researchers accustomed to paper-based documentation. These insights align with the Technology Acceptance Model [5,13], confirming that perceived ease of use and usefulness are decisive for adoption. Similarly, Higgins et al. [2] highlight that early involvement of users and minimizing additional administrative burdens are key for successful ELN implementation.

The pilot phase also revealed cultural and organizational barriers such as resistance to change, uncertainty regarding data migration, and varying levels of digital readiness. To support the transition process, a multifaceted training and support program consisting of hands-on workshops, user manuals, an online helpdesk, and individual consultation sessions was introduced. Targeted measures helped to build trust, reduce entry barriers, and increase system acceptance among different user groups.

The findings of the pilot phase directly informed the development of onboarding materials, metadata standardization strategies, training formats, governance considerations, and migration-support tools used during the subsequent institutional rollout.

By systematically addressing these challenges, the pilot phase provided practical indications regarding the operational feasibility of eLabFTW and informed the preparation of a faculty-wide rollout. It demonstrated that ELN implementation is not solely a technical task but also an opportunity to consolidate documentation practices and promote the FAIR principles [2,6].

### Technical implementation

The technical implementation of eLabFTW was realized through close collaboration between the Faculty of Medicine, the UKH, and central IT service units. The institutional backbone is provided by the Information and Communication Technology service (IUK), which operates the core IT infrastructure for both the UKH and the faculty. This ensured that the new system could be deployed securely, reliably, and in compliance with institutional and regulatory requirements.

The Data Integration Center (DIC) assumed responsibility for the installation, maintenance, and long-term operation of eLabFTW. The DIC serves as the central interface between clinical care and medical research and coordinates data management activities with various departments, including medical informatics, clinical documentation, and research centers. This structure guarantees standardized, privacy-compliant collection, processing, and use of research data for both clinical and academic contexts.

Due to the overlap of technical and methodological expertise, the DIC collaborates closely with academic institutes such as the Institute of Medical Epidemiology, Biometry, and Informatics (IMEBI) to support data integration, analysis, and digitally assisted research.

A key component of the infrastructure is Keycloak [14], an open-source identity and access management solution implemented by the DIC. Keycloak provides secure and flexible user authentication based on the Security Assertion Markup Language (SAML), ensuring compliance with institutional data protection requirements. The DIC continues to maintain and update this system, providing a reliable foundation for managing access to sensitive research data.

The eLabFTW environment is structured into three independent Linux-based instances: a production instance, a quality assurance instance, and a test instance. This multi-instance architecture enables secure deployment and controlled rollout of updates. New features and configurations are first evaluated in the test environment, verified in the quality assurance instance, and only then transferred to the production system. This staged process minimizes the risk of downtime, data loss, or instability.

Technical operation and support are jointly managed by the DIC and IUK, combining expertise in research data management and infrastructure administration. Furthermore, eLabFTW has been fully integrated into the institutional storage and archiving systems, ensuring compatibility with existing workflows. This integration allows researchers to efficiently store and retrieve their data and promotes adherence to best practices in data stewardship [15]. By embedding eLabFTW within the broader institutional IT framework, the implementation supports scalability, security, and sustainability for future digital research infrastructures.

## Results of the empirical investigation of the current situation

### Design and methodology

An online survey (LimeSurvey [16]) comprising 28 questions was administered between November 2024 and March 2025. It covered demographics, current documentation methods, satisfaction, challenges, security measures, and expectations for an ELN. Logical branching ensured that participants answered only relevant questions; subsequent items were displayed conditionally based on previous responses.

The survey was originally conducted in German, as it targeted researchers at a German academic institution. For publication purposes, all survey items were translated into English by the authors to ensure conceptual consistency.

The survey distinguishes between related but context-dependent concepts such as “research documentation”, “research data”, and “research results”. These terms reflect different stages and aspects of the research process (e.g., raw data, processed results, and overall documentation practices). For the purpose of this study, these concepts are considered complementary and are analyzed in an integrated manner.

### Data processing and visualization

Descriptive statistics and time-based evaluations of the survey data were performed using R, with R Shiny [17] supporting interactive multi-response analyses and exploratory visualization. Platform usage statistics extracted from the production database were analyzed using R and visualized in Apache Superset [18]. Interim analyses supported early pattern detection and dynamic data exploration.

### Population and scope

The survey was started by 58 participants; 55 completed it. All analyses reported here are based on these 55 complete responses (100%). Conditional presentation logic was applied throughout the questionnaire to ensure that participants only answered relevant items. As part of this logic, respondents were separated into two subgroups according to their prior experience with laboratory documentation. Three participants were classified as having *no prior experience* and were subsequently guided through a shortened question path starting from item G00Q03 (see Supplementary Table S3 in S1 Appendix). Due to the very small sample size and the resulting risk of potential re-identification, this subgroup is not considered in the analyses presented in this publication.

Consequently, subgroup-based analyses presented in the Results section refer exclusively to participants with prior documentation experience (n = 52). The detailed list of survey items and additional tables not reproduced in the main text are provided in the S1 Appendix (Survey Item Catalogue and Supplementary Survey Results).

### Survey results

#### Documentation practices.

The survey participants represented a broad spectrum of research staff within the medical and life sciences disciplines. The respondent group included professors (15%), postdoctoral researchers (incl. habilitands) (25%), laboratory heads (15%), and various categories of technical and scientific personnel. This diversity of perspectives provided a representative overview of documentation practices across laboratory environments. The survey was intended to capture a broad range of perspectives across different research environments and career stages rather than to provide a fully representative institutional census.

As shown in Table 1, the largest group comprised postdoctoral researchers, followed by professors and laboratory heads. The table presents a clustered representation of participant roles; the complete list of single-choice response options is provided in Supplementary Table S2 in the S1 Appendix. The composition reflects the institutional structure, in which research groups typically consist of a mix of senior academics and early-career scientists.

**Table 1:** Distribution of Participants by Professional Role (Survey item G00Q01).

Role	# Responses	Percentage
Postdoctoral researchers (postdocs & habilitands)	14	25.46%
Laboratory heads	8	14.55%
Professors	8	14.55%
Doctoral candidates (PhD students)	8	14.55%
Science-supporting staff	7	12.73%
Scientific staff	7	12.73%
Other individual roles	3	5.46%

*Note:* Categories with fewer than three responses were aggregated as “Other individual roles” to maintain participant anonymity. The corresponding survey item and original response options are documented in the Supporting Information.

In terms of disciplinary affiliation, about two thirds of respondents were active in clinical and medical research, while approximately 15% worked in pharmaceutical sciences and another 15% in biochemistry. Interdisciplinary areas such as bioinformatics and data science accounted for roughly 5%, underscoring the range of the scientific landscape represented.

Traditional documentation methods continue to dominate everyday research practice, as summarized in Table 2. Electronic spreadsheets, most commonly Microsoft Excel, were reported by nearly 87% of respondents, closely followed by paper-based laboratory notebooks (85%) and word-processing programs (71%). Approximately one in four participants reported using additional, often project-specific tools. These findings indicate that hybrid documentation models – combining analog and digital components – remain prevalent.

**Table 2:** Current Methods Used for Research Documentation (Survey item G01Q01).

Method	# Responses	Percentage
Electronic spreadsheets (e.g., Excel)	45	86.54%
Paper laboratory notebooks	44	84.62%
Word-processing programs (e.g., Word)	37	71.15%
Additional tools	15	28.85%

*Note:* Multiple responses were permitted; therefore, percentages do not sum to 100%.

**Fig 1:**
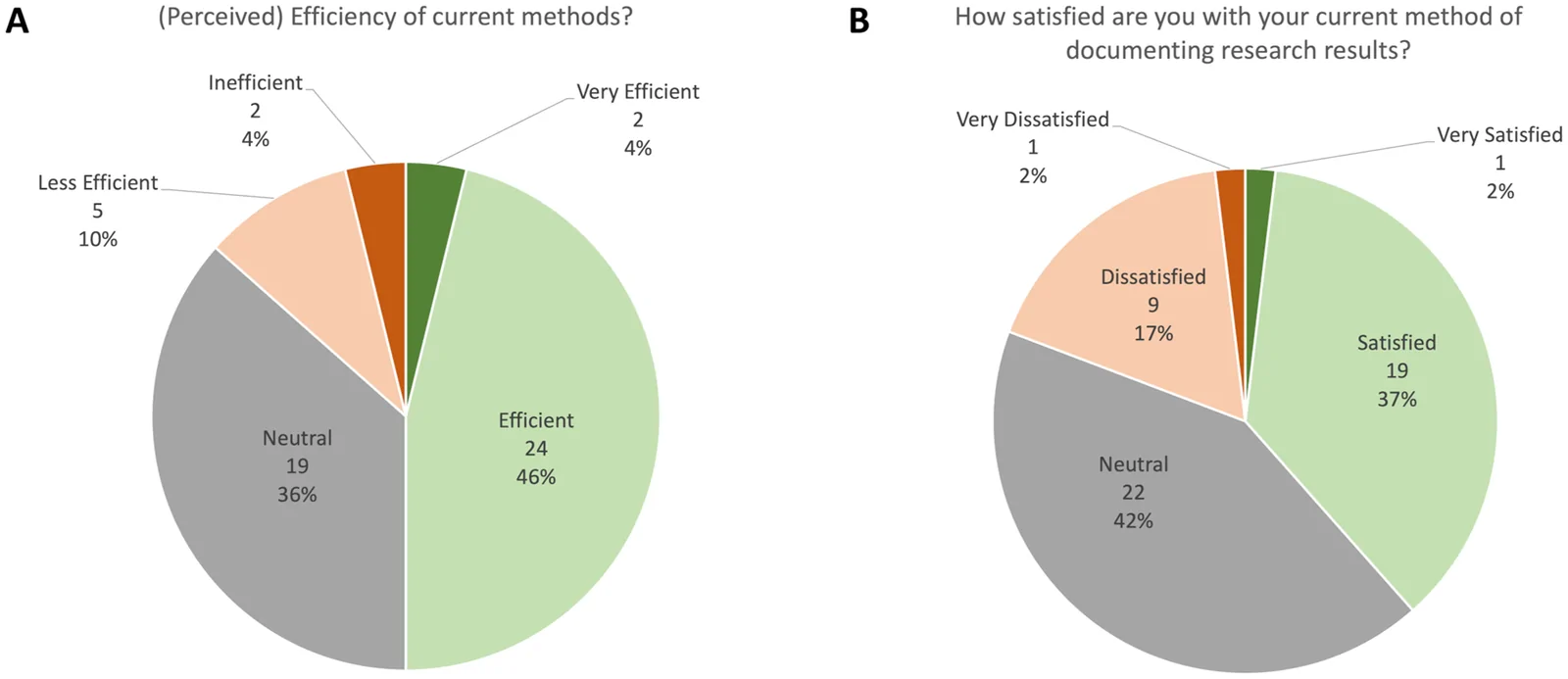
Perceived efficiency and satisfaction with current documentation methods. (A) Distribution of responses regarding the perceived efficiency of current research documentation practices. (B) Overall satisfaction with current documentation methods as reported by survey participants.

However, as shown in Fig 1 perceptions of efficiency were mixed. Fewer than 4% of respondents described their current methods as “very efficient,” while most (around 46%) considered them only “efficient” or “neutral.” This reflects a general dissatisfaction with current workflows and suggests room for improvement.

Satisfaction levels followed a similar pattern. Only about 2% reported being very satisfied with their documentation processes, while roughly 37% were satisfied and 42% expressed neutral views. Around one fifth were dissatisfied or very dissatisfied. This distribution illustrates a moderate but widespread sense of improvement potential among researchers.

**Fig 2:**
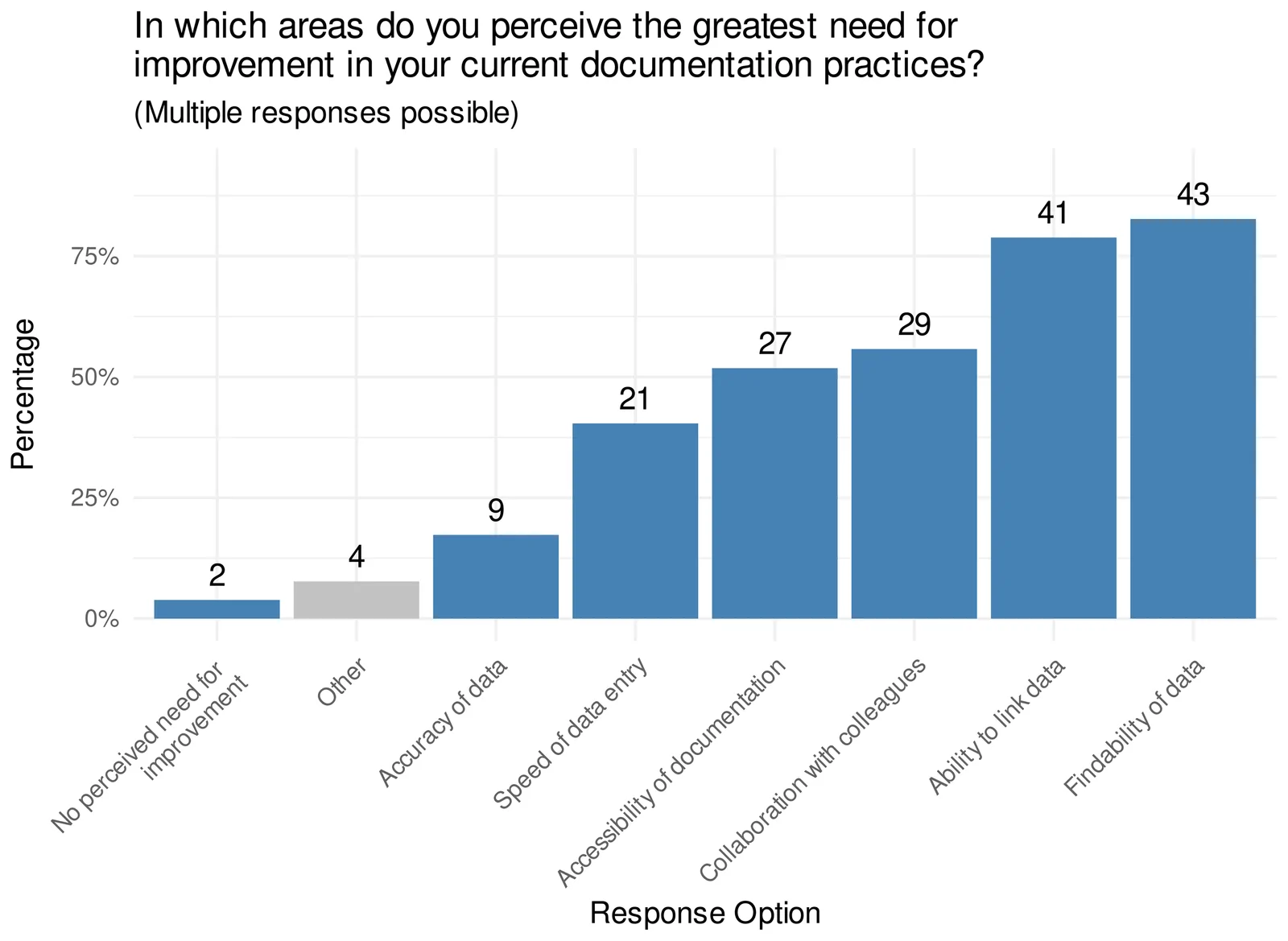
Key areas identified for improving current documentation practices. The figure summarizes the most frequently cited needs for improvement, including data findability, data linkage, collaboration, accessibility, speed of data entry, and data accuracy. Multiple responses were permitted.

When asked to identify specific areas in need of improvement, respondents most frequently mentioned the findability of data (83%), the ability to link related information (79%), and collaboration with colleagues (56%). Accessibility (52%) and speed of data entry (40%) followed, while only 17% cited data accuracy as a major concern. As shown in Table 3 and Fig 2, these findings highlight that organizational and structural challenges – rather than data quality itself – represent the principal bottlenecks.

**Table 3:** Key Areas Identified for Improving Current Documentation Methods (Survey item G01Q06).

Improvement Area	# Responses	Percentage
Findability of data	43	82.69%
Ability to link data	41	78.85%
Collaboration with colleagues	29	55.77%
Accessibility	27	51.92%
Speed of data entry	21	40.38%
Accuracy of data	9	17.31%
Other	4	7.69%
No perceived need for improvement	2	3.85%

*Note:* Multiple responses were allowed. The category “Other” is shown in grey for completeness.

Overall, these results demonstrate that researchers at the University Medicine Halle possess substantial documentation experience but rely primarily on traditional tools. They also share a clear interest in improving data organization, linking, and collaborative workflows, which provides a strong basis for introducing structured electronic systems.

#### Security, access, and expectations.

Security practices among participants varied across experience levels, as summarized in Table 4. Regular backups (58%) and password protection (54%) were the most frequently reported measures, whereas approximately one quarter of respondents (23%) indicated that they used no dedicated security measures. Participants with longer documentation experience more frequently reported the use of password protection and regular backups, whereas less experienced subgroups more often reported the absence of formalized security practices. Although subgroup sizes for early-career participants were small, the findings indicate the importance of institutional guidance, standardized onboarding, and awareness-building regarding research data security and access management.

**Table 4:** Adoption of Security Practices by Experience Level.

Experience Level	Password Protection	Physical Security	Regular Backups	No Measures
Less than 1 year (n = 1)	0 (0%)	0 (0%)	0 (0%)	1 (100%)
1–3 years (n = 5)	0 (0%)	0 (0%)	0 (0%)	5 (100%)
3–5 years (n = 3)	2 (67%)	1 (33%)	2 (67%)	0 (0%)
More than 5 years (n = 43)	26 (60%)	15 (35%)	28 (65%)	6 (14%)

*Note:* Only participants with prior documentation experience were included in subgroup analyses (n = 52). Participants reporting no prior documentation experience (n = 3) were excluded due to conditional survey branching. Percentages refer to the proportion of respondents within each subgroup. Multiple responses were permitted for selected questions; percentages therefore do not sum to 100%.

Ease of access to existing documentation emerged as another challenge, as detailed in Supplementary Table S9 in the S1 Appendix. Almost half of the respondents (48%) described access as easy, 38% remained neutral, and about 10% found access difficult. When collaboration and data exchange were assessed, only one fifth rated support as good, whereas more than 40% considered it poor or very poor (Supplementary Table S10 in the S1 Appendix). These findings indicate that collaboration and structured information exchange remain important limitations of current documentation practices.

**Fig 3:**
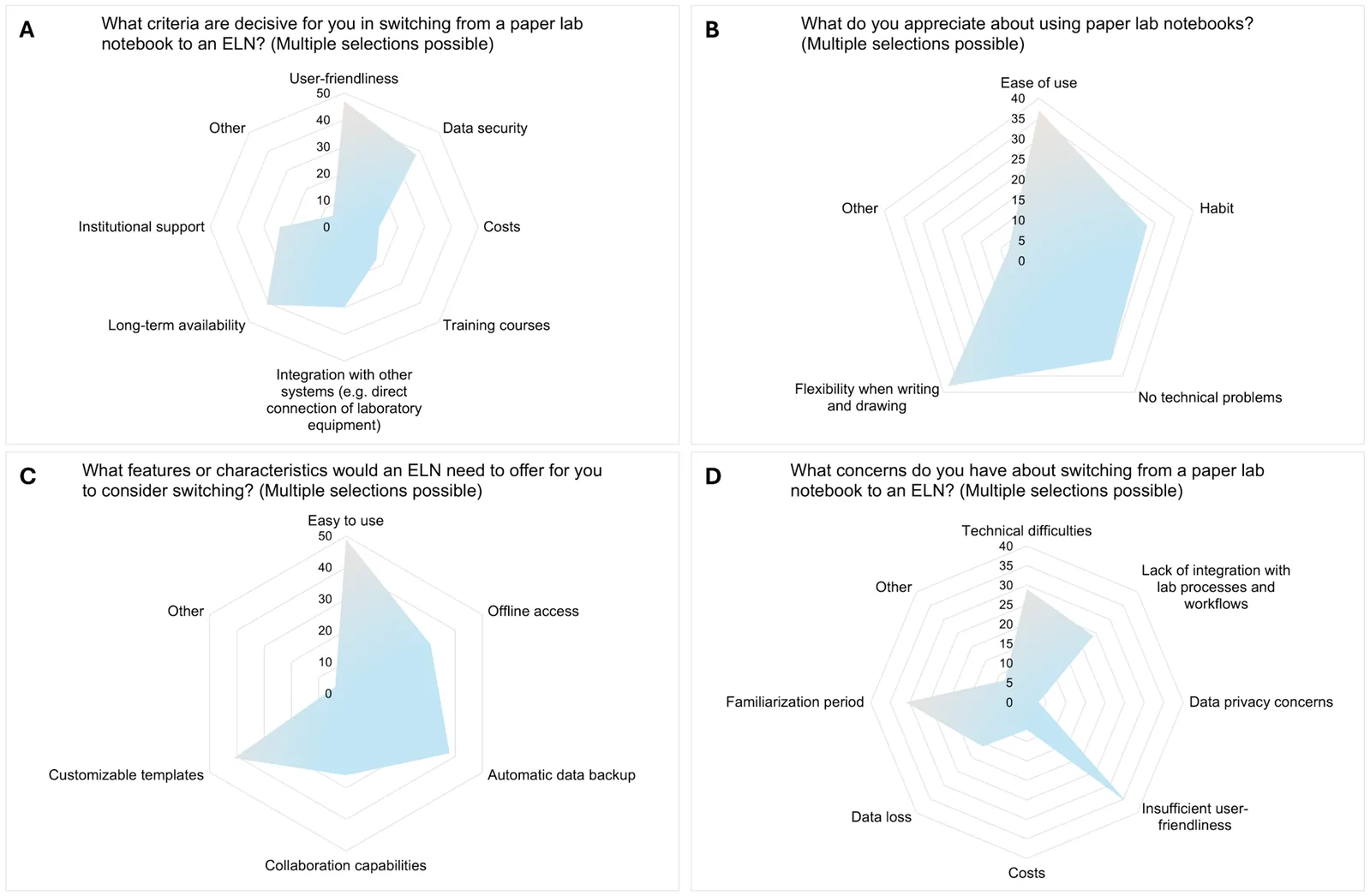
Assessment of criteria, benefits, features, and concerns related to electronic laboratory notebooks. Composite figure consisting of four radar charts summarizing participant responses: (A) Criteria considered decisive when switching from a paper lab notebook to an electronic laboratory notebook (ELN). (B) Aspects participants appreciate about paper-based lab notebooks. (C) Features or characteristics an ELN would need to offer for participants to consider switching. (D) Concerns associated with switching from a paper lab notebook to an ELN. Multiple selections were possible for all questions.

When asked about desired improvements and key features for an electronic system, respondents clearly prioritized usability and reliability. As shown in Table 5 and visually summarized in Fig 3 (A), user-friendliness was selected by 47 respondents (90%), long-term data availability by 41 (79%), and data security by 38 (73%). Integration with existing systems received 30 selections (58%), and institutional support 24 (46%), while cost was mentioned less frequently with 13 responses (25%).

**Table 5:** Criteria Considered Most Important for Adopting an Electronic Laboratory Notebook (Survey item G01Q12).

Criterion	# Responses	Percentage
User-friendliness	47	90.38%
Long-term data availability	41	78.85%
Data security	38	73.08%
Integration with other systems	30	57.69%
Institutional support	24	46.15%
Training offers	17	32.69%
Cost	13	25.00%
Other	6	11.54%

*Note:* Multiple responses were permitted; percentages therefore do not sum to 100%.

The most frequently cited barriers to ELN adoption were insufficient user-friendliness, reported by 36 respondents (69%), the effort of familiarization (31; 60%), and potential technical difficulties (29; 56%). These patterns are detailed in Supplementary Table S15 in the S1 Appendix and depicted in Fig 3(D). A smaller proportion mentioned lack of integration with laboratory processes (24; 46%), while issues such as data loss (16; 31%) or privacy concerns (3; 6%) occurred less frequently. These results suggest that perceived usability and workflow compatibility are more decisive than security fears or cost barriers. The importance of appropriate institutional support was also evident. As shown in Table 6, over 70% of respondents considered structured introduction courses essential for a successful transition, followed by continuous technical assistance (52%) and accessible user manuals (46%). Roughly one third mentioned the usefulness of office hours and peer-to-peer exchange groups, underscoring the need for proactive, guided onboarding rather than passive self-learning. Additional response patterns related to current documentation practices – such as flexibility (73%) and user-friendliness (71%) of paper notebooks – as well as desired ELN features including customizable templates (79%) and automatic data backup (73%) are summarized in the respective tables and visualized in Fig 3 (B-C).

**Table 6:** Types of Support Considered Important for Transitioning to an ELN (Survey item G01Q16).

Support Type	# Responses	Percentage
Introduction courses	37	71.15%
Continuous technical support	27	51.92%
User manuals	24	46.15%
Office hours	22	42.31%
Peer-support groups	18	34.62%
Other	5	9.62%

*Note:* Multiple responses were permitted; percentages therefore do not sum to 100%.

**Fig 4:**
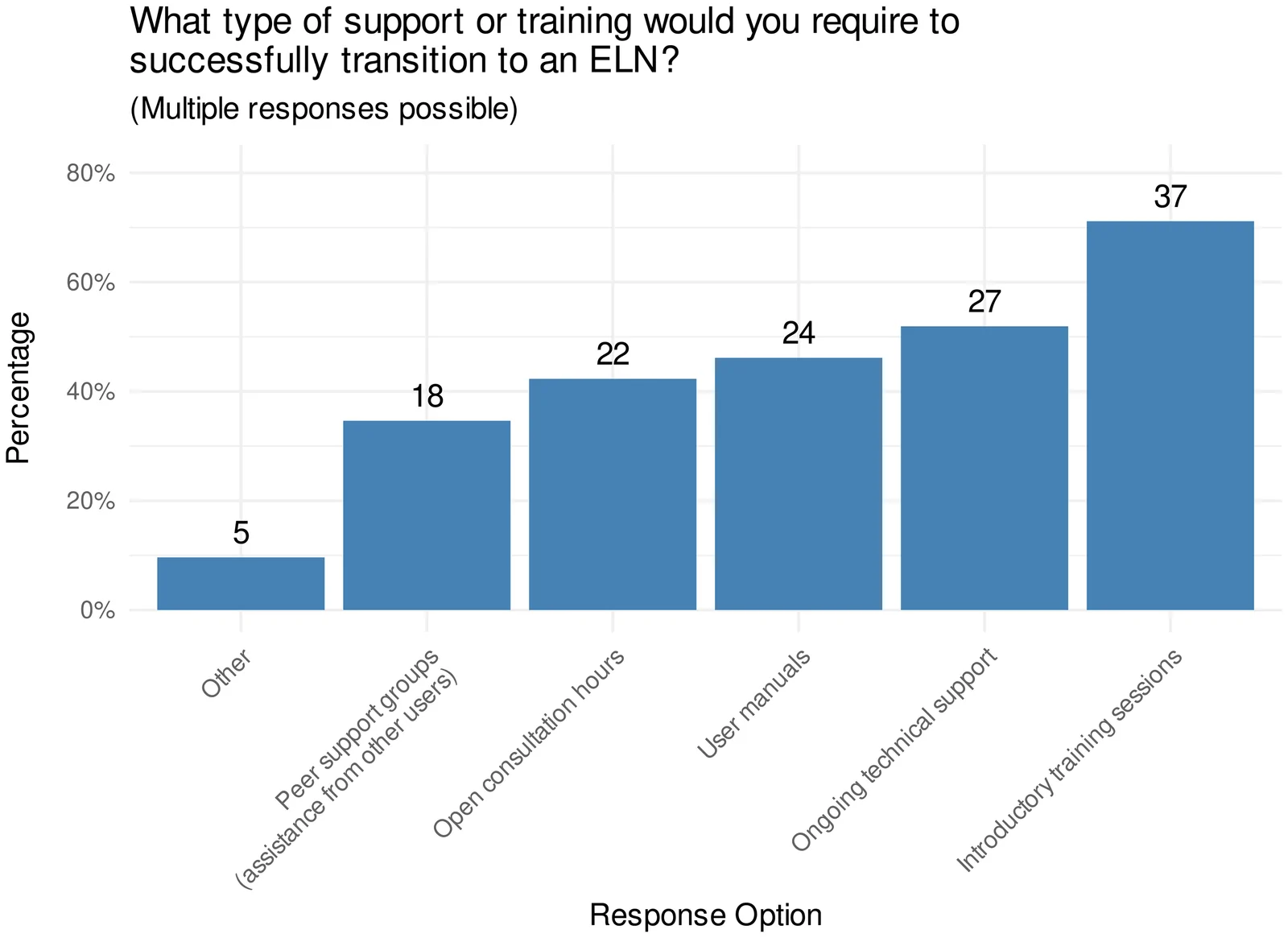
Preferred types of institutional support for ELN implementation. Distribution of responses indicating which forms of training and support participants consider most important when transitioning to an electronic laboratory notebook.

Fig 4 provides a graphical overview of the distribution of preferred support formats summarized in Table 6. Introductory training sessions were selected most frequently, followed by ongoing technical support and the availability of user manuals. Open consultation hours and peer-support groups were mentioned by a smaller, but still substantial proportion of respondents, while only few participants selected other forms of support. Overall, the bar plot illustrates a clear ranking of support preferences and highlights the relative prominence of structured and continuous support formats compared to more informal options.

To better understand these preferences, participants were also grouped according to their years of experience. The trends remained consistent: more experienced users emphasized ongoing technical support, while early-career scientists favored introductory training and clear documentation.

Taken together, these findings reveal a well-defined but nuanced picture: most researchers at the University Medicine Halle demonstrate readiness to adopt electronic laboratory notebooks, provided that usability, security, and institutional guidance are ensured. The combination of practical motivation, explicit support needs, and positive baseline engagement forms a strong foundation for sustainable ELN implementation. Detailed response distributions are presented in the “Supplementary Survey Results” section of the S1 Appendix.

### Platform adoption and usage during institutional rollout

During the 22-month institutional rollout period (October 2024 to July 2026), the platform gained 232 new registered users, corresponding to an average of 10.5 new users per month. By the end of the observation period, the platform comprised 237 registered users across 32 research teams. Users had created 4,263 active experiments and 9,624 active resources, corresponding to an average of 45.3 new experiments and 103.4 new resources per week during the rollout period. These platform usage statistics complement the survey findings by providing objective indicators of institutional adoption and routine platform use.

## Discussion

### Current documentation landscape and adoption factors

The results of this study combine survey-derived user perspectives with objective platform usage metrics, providing complementary insights into both implementation requirements and platform adoption during institutional rollout. Together, these findings suggest that the identified implementation strategy successfully translated initial user requirements into sustained institutional uptake. The survey findings reveal a complex and heterogeneous landscape of laboratory documentation practices, particularly with respect to existing documentation workflows and the requirements for introducing an electronic solution. While most researchers possess substantial experience in documenting scientific data, the results indicate recurring usability challenges in current documentation workflows rather than uniformly low efficiency. Time consumption and difficulties in data organization and in finding information were reported most frequently, whereas concerns about data security were mentioned less often. Overall efficiency ratings were predominantly neutral to positive, with nearly half describing their approach as efficient and more than one third reporting a neutral assessment. Only a small minority rated their methods as less efficient or inefficient. Traditional paper notebooks and electronic spreadsheets continue to dominate, demonstrating both the persistence of established habits and the lack of standardized digital infrastructure. This situation mirrors broader trends in the scientific community, where documentation quality is acknowledged as critical for reproducibility, yet modernization proceeds unevenly [2,6]. The data confirm that usability and efficiency are decisive for user acceptance, consistent with the technology acceptance framework proposed by Davis et al. [5]. These findings are consistent with earlier empirical evidence from a comparative PLOS ONE study, which demonstrated that researchers’ acceptance of electronic laboratory notebooks is strongly influenced by perceived usability, searchability, and workflow fit rather than by technical feature completeness alone [19]. In that study, general-purpose documentation tools and dedicated ELN systems were valued differently across user groups, underscoring that successful ELN implementation requires careful alignment with existing documentation practices and user expectations rather than a one-size-fits-all approach. Although many researchers recognize the potential advantages of ELNs, adoption is frequently hindered by practical barriers such as insufficient user-friendliness, the anticipated familiarization period, and concerns about technical difficulties and integration with established laboratory processes. Importantly, respondents also expressed uncertainty about migrating existing documentation and legacy systems into a new ELN and the risk of data loss during this transition, a theme that was likewise recurrent during the initial onboarding and exchange events. Cost considerations played a comparatively minor role, and data privacy concerns were reported only rarely.

### Survey-informed onboarding framework

To ensure that the implementation strategy was aligned with actual user needs, the onboarding process was developed iteratively based on pilot experiences, structured user feedback, and observations from early implementation activities. Initial pilot phases and onboarding events revealed recurring practical barriers, particularly regarding usability, migration of existing documentation structures, and the integration of ELN workflows into established laboratory routines. These observations were subsequently supported and further differentiated by the structured user survey.

The survey demonstrated that usability (90%) and training requirements (71%) were among the most important factors influencing the adoption of an electronic laboratory notebook. These findings directly informed the development of structured onboarding measures, including guided workshops, user manuals, onboarding checklists, and continuous support formats. Furthermore, challenges related to data organization (63%) and information retrieval (63%) highlighted the importance of standardized templates and structured metadata practices within the ELN environment. Many research groups also relied on extensive spreadsheet-based resource collections, including inventories, SOPs, project structures, antibodies, primers, and device lists, which represented a substantial migration challenge during onboarding.

To reduce these barriers, dedicated migration and harmonization tools such as the Excel-to-CSV Converter and the CSV/SQL Merger were developed to support the reuse of existing documentation structures and facilitate the transition toward standardized ELN-supported workflows.

**Fig 5:**
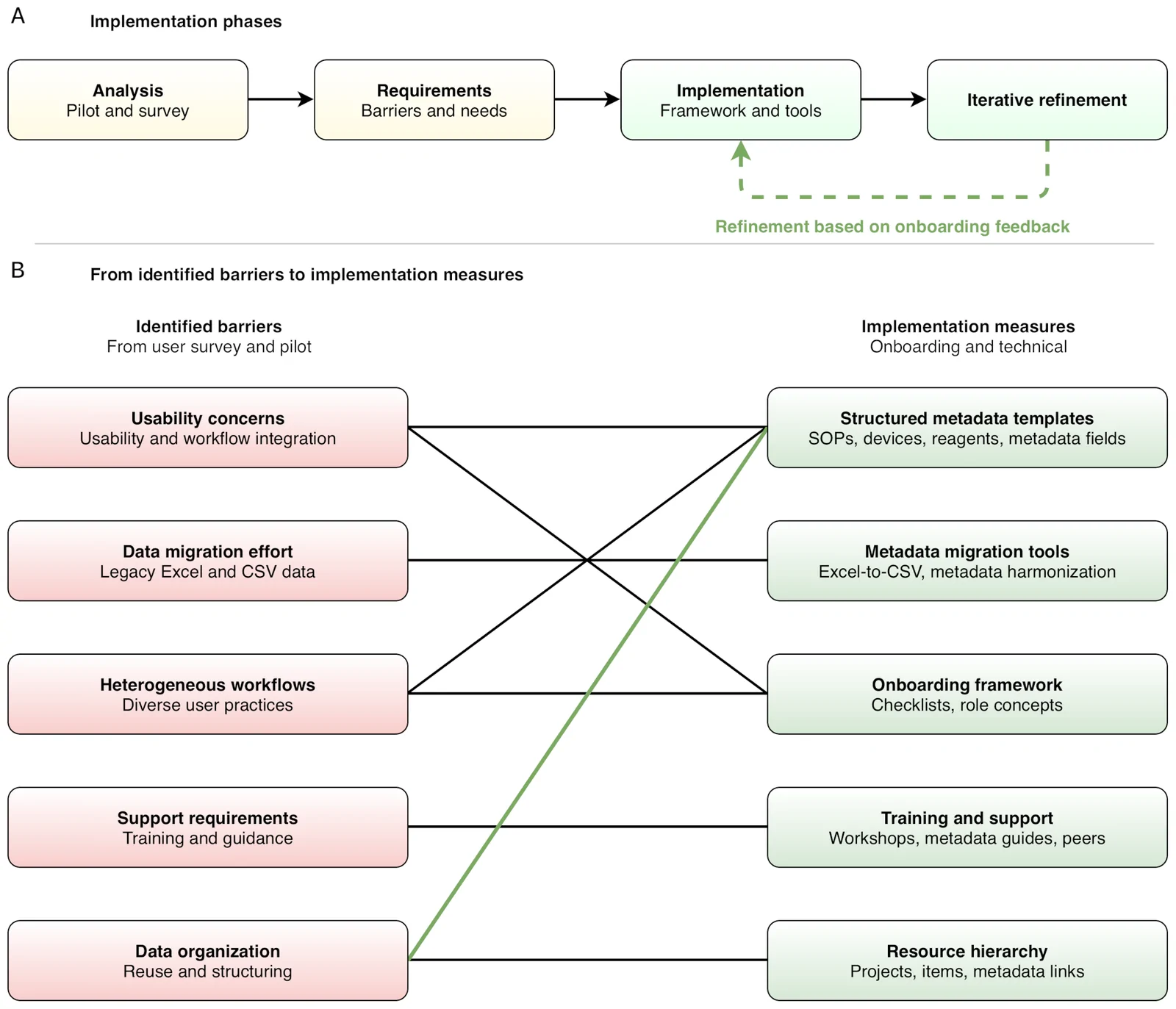
Survey-informed implementation and onboarding framework for institutional ELN adoption. (A) Iterative implementation process integrating pilot experiences, user surveys, onboarding activities, and continuous refinement based on user feedback. (B) Mapping of identified user barriers and requirements to corresponding onboarding and technical implementation measures, including metadata standardization strategies, migration-support tools, structured templates, and training concepts developed during the introduction of eLabFTW at University Medicine Halle.

Overall, this survey-informed and iterative approach ensured that the implementation strategy was not purely technology-driven but grounded in the practical needs, expectations, and existing documentation realities of the users. Taken together, these findings indicate that ELN adoption should not be understood as a single technical deployment step, but as an iterative onboarding and implementation process. Initial pilot experiences, survey-derived user requirements, and feedback from early onboarding activities jointly informed the development of the implementation framework shown in Fig 5.

The framework illustrates how identified barriers and practical user requirements were translated into targeted onboarding and technical implementation measures. In particular, usability concerns, heterogeneous documentation practices, migration challenges, and support requirements informed the development of structured metadata templates, migration tools, onboarding concepts, and training strategies. The framework further emphasizes the iterative refinement of implementation measures based on continuous user feedback during early adoption phases. The framework highlights that successful ELN implementation depended not only on technical deployment, but also on structured onboarding, metadata standardization, and continuous adaptation to existing laboratory workflows and user practices.

### Training needs, security practices, and institutional support

Respondents expressed a strong preference for structured onboarding. More than two thirds favored formal introduction courses and continuing support, and many valued access to manuals and peer learning. These expectations align with the implementation approach used for eLabFTW at the University Medicine Halle, which emphasizes guided onboarding, sandbox testing, and direct user consultation. As Higgins et al. [2] have shown, active engagement and early feedback mechanisms are crucial for overcoming skepticism and establishing trust in digital tools. Such measures transform ELN introduction from a top-down IT project into a collaborative capacity-building exercise.

The security data reveal a notable divide between experienced and less experienced researchers. Those with over five years of documentation practice tend to apply multiple safeguards, while novices rarely use any. This finding suggests that awareness of data protection grows with professional maturity but is not systematically trained. Institutions can close this gap by embedding minimum standards for digital documentation and by offering targeted instruction. The integration of Keycloak-based authentication and centralized data management, as described in the technical implementation section, directly addresses these requirements and illustrates how technical system design can support structured and secure documentation workflows. At the same time, collaborative access had to be balanced with institutional security requirements. Although the platform is hosted within the secure infrastructure of University Medicine Halle, access was intentionally extended beyond research groups within University Medicine Halle to research groups at Martin Luther University Halle-Wittenberg participating in joint research projects with University Medicine Halle. This enables collaborative documentation across institutional boundaries while maintaining centralized authentication and role-based access management. In this context, role-based access management should be distinguished from broader data security measures such as backup strategies, authentication infrastructure, and long-term data protection.

The survey results indicate that security and usability represent distinct dimensions of documentation practice. While many respondents reported using security-related measures such as backups or password protection, usability-related aspects were more prominent when participants evaluated a potential transition to an ELN. User-friendliness was the most frequently selected criterion for switching to an ELN, and insufficient user-friendliness was also the most frequently reported concern regarding such a transition.

This suggests that existing security practices do not necessarily reduce the importance of usability for adoption decisions. Rather, participants appeared to distinguish between measures used to protect current documentation and practical requirements that determine whether a new documentation system can be integrated into daily laboratory workflows.

A further insight concerns the cultural dimensions of change. As noted in the literature, data ownership and the reluctance to share results remain pervasive barriers in scientific communities [8]. Several free-text responses from the present survey reflect similar attitudes, particularly the perception that electronic documentation could expose ongoing work to external monitoring. Tackling these concerns requires not only secure infrastructures but also institutional policies that reward transparency and collaboration. The implementation of eLabFTW thus represents an opportunity to promote FAIR-compliant documentation practices [6], supported by explicit institutional commitment and structured implementation measures.

### Transition to digital workflows and integration into research data management (RDM) ecosystems

The interplay of traditional and digital tools also highlights the transitional nature of the current documentation environment. While paper notebooks offer familiarity and flexibility, they fail to meet demands for collaboration, searchability, and integration. The high frequency of documentation updates – daily or weekly for most participants – indicates readiness for a digital platform that preserves these habits while adding automation and structure. eLabFTW fulfills these conditions by providing user-defined templates, searchable metadata, and controlled sharing, thereby combining flexibility with standardization.

The reported challenges – time consumption, disorganization, and difficulty retrieving information – point to systemic inefficiencies that can be addressed by ELNs. In this context, the success of digital transformation depends less on the availability of sophisticated features and more on the perceived improvement of everyday workflows. The positive response to features such as automatic backups, customizable templates, and collaboration tools indicates that researchers primarily seek solutions that simplify routine tasks rather than overhaul them. This pragmatic orientation should guide future development and communication strategies.

The results also suggest that institutional frameworks strongly influence acceptance. Where users perceive long-term infrastructure reliability and accessible technical support, confidence in digital systems increases substantially. The collaboration between the Data Integration Center, the IT services, and research departments provides an example of how such stability can be achieved. In particular, the multi-instance server architecture for eLabFTW ensures operational reliability and compliance, which in turn reinforces user trust.

Finally, the survey underscores the importance of connecting ELNs with broader research data management initiatives. As part of the FAIR principles, ELNs should not be isolated documentation tools but integral components of interoperable data ecosystems.

A central aspect repeatedly emphasized during onboarding and training activities was that the scientific value of an ELN does not primarily arise from storing uploaded files alone, but from the structured representation of experimental information. Simple document uploads without standardized metadata or searchable parameters risk creating isolated “data graves” with limited long-term usability.

Consequently, onboarding focused strongly on the use of structured templates, predefined metadata fields, standardized units, and controlled vocabularies. In eLabFTW, this was implemented through configurable extra fields, structured numeric metadata fields with predefined units, and reusable dropdown dictionaries. These structures support more consistent documentation practices, reduce ambiguity and typographical variation, and improve the findability and reusability of experimental information within institutional workflows. The integration of eLabFTW with dashboard tools such as Apache Superset [18] and Confluence [20] demonstrates how documented information can be transformed into actionable knowledge (see supplemental information S4 in S1 Appendix). These extensions increase both the scientific and organizational value of the ELN by enabling the secondary use of structured documentation data through interactive dashboards supporting quality monitoring, reporting, and institutional oversight [15].

A further component supporting the transition toward digital documentation was the development of institution-specific R Shiny applications that facilitate data preparation during onboarding and initial setup. These tools emerged from practical needs identified both in the pilot phase and throughout the onboarding of the first research teams. Many laboratories relied on extensive Excel-based catalogues, inventories, and sample lists that were unsuitable for direct import into the ELN.

This challenge was particularly relevant during the initial setup of ELN resources. Research groups often maintained extensive Excel-based lists of devices, consumables, chemicals, projects, SOPs, antibodies, primers, and other laboratory resources. Manually recreating these structures in eLabFTW would have required substantial effort and could have increased the risk of inconsistent metadata entry.

To address this practical barrier, the Excel-to-CSV Converter was developed as a dedicated onboarding tool. It allows users to transform existing spreadsheet structures into eLabFTW-compatible CSV import files while preserving references to the original data sources. By enabling column mapping, metadata standardization, and structured export, the tool reduces manual work during initial resource setup and supports a smoother transition from decentralized spreadsheet-based documentation to structured ELN-supported workflows. The implementation process further indicated that resistance to ELN adoption was often associated less with fundamental opposition to digital documentation than with concerns regarding migration effort, usability, additional workload, and disruption of established laboratory routines. The application additionally supports the extraction of controlled vocabularies and reusable metadata dictionaries from existing spreadsheet structures, facilitating the generation of standardized ELN fields and reducing inconsistencies caused by heterogeneous naming practices.

Together with the CSV/SQL Merger, these tool-supported approaches facilitate data migration and metadata harmonization by providing graphical, browser-based interfaces for preprocessing existing datasets and exporting structured files compatible with the eLabFTW import format. In this way, the applications act as transitional tools that translate heterogeneous legacy data into interoperable structures, thereby reducing onboarding barriers and supporting FAIR-oriented documentation practices. Both tools were implemented as interactive R Shiny applications (R version 4.2.1) using established packages for web application development, data processing, and export functionality [17,21–26]. They are publicly available as open-source software via GitHub and archived on Zenodo (Excel-to-CSV Converter: DOI 10.5281/zenodo.15533906; CSV/SQL Merger: DOI 10.5281/zenodo.15533992). Extended technical documentation, including usage instructions, screenshots, and metadata mapping examples, is provided in the Supporting Information accompanying this article. Making these implementation resources openly available supports transparency, reproducibility, and reuse of the presented onboarding workflow by other institutions.

In sum, the findings suggest that the successful implementation of an Electronic Laboratory Notebook requires a comprehensive strategy encompassing user engagement, technical robustness, and institutional commitment. Cultural factors such as openness, trust, and collaboration are just as vital as software configuration. The experience at the University Medicine Halle illustrates that, when these dimensions are aligned, digital documentation systems like eLabFTW can serve as catalysts for sustainable, transparent, and efficient research practices.

### Limitations

The findings presented in this study should be interpreted in light of several limitations related to study design, scope, and transferability.

First, the survey data are based on self-reported perceptions rather than objective performance metrics. Assessments of efficiency, usability, security practices, and adoption barriers therefore reflect subjective experiences shaped by individual expectations, prior familiarity with documentation tools, and established working routines. While such perception-based data are highly relevant for understanding user acceptance and decision-making processes [5,13], they do not allow direct conclusions regarding measurable improvements in documentation quality, time efficiency, or error reduction. Similar limitations of perception-based survey data have been reported in prior large-scale studies on research data practices and data sharing behavior [27]. Given the exploratory design, the multiple-response structure of several survey items, and the small size of some subgroups, analyses were restricted to descriptive statistics to avoid overinterpretation of subgroup differences.

Second, although the study was conducted primarily at the University Medicine Halle, the participant group encompassed a heterogeneous set of laboratory-based research environments, including groups from biochemistry and pharmacy. This diversity increases the internal breadth of perspectives captured by the survey but does not remove contextual constraints. Institutional governance structures, available technical support, and local documentation cultures are known to influence both perceived challenges and adoption dynamics of electronic documentation systems [2,28]. Consequently, while many identified patterns align with observations reported in the broader literature on research data management practices [27,29], their relative importance and practical implications may vary across institutions and disciplinary contexts.

Third, the results reflect attitudes and practices during an early phase of ELN consideration and implementation. Accordingly, the present study focuses on user requirements, implementation, and early platform adoption. A detailed evaluation of post-implementation user experiences, including perceived collaboration after routine use, is beyond the scope of the present manuscript. Previous studies have shown that perceptions of usability, integration, and usefulness of digital research infrastructure can evolve substantially over time as users gain experience and workflows stabilize [15,30]. Long-term effects on documentation quality, collaboration, reproducibility, and user acceptance therefore require dedicated longitudinal evaluation.

Finally, participation in both the survey and the accompanying onboarding and exchange events was voluntary. This introduces the possibility of self-selection bias, as researchers with a stronger interest in digital tools or research data management may be overrepresented. Comparable limitations related to participation bias and disciplinary self-selection have been described in survey-based studies on data practices in academic research [27,29].

Despite these limitations, the study provides empirically grounded insights into practical barriers and success factors associated with the introduction of electronic laboratory notebooks in a complex academic research environment. By situating institution-specific findings within established acceptance models and the broader literature on research data management [5,6], the results offer a transparent and context-aware contribution while clearly delineating the boundaries of their applicability and generalization.

## Conclusion

The survey findings and subsequent implementation experiences suggest that successful ELN adoption requires not only appropriate technical infrastructure but also structured onboarding, metadata standardization, training, and continuous user support. The analysis at the University Medicine Halle revealed that while analog methods remain widespread, there is a clear willingness among researchers to adopt structured digital solutions once usability, reliability, and institutional support are ensured.

The introduction of eLabFTW, combined with user-centered onboarding and sustained technical assistance, was designed to support the transition between individual user expectations and organizational infrastructure requirements. The approach emphasizes that an electronic lab notebook is not just an administrative or technical tool, but a strategic component of modern research data management. It can support traceability, facilitate collaboration, and contribute to FAIR-oriented data management practices [6].

Long-term sustainability of ELN infrastructures requires not only stable technical operation but also strategies for future data migration, interoperability, and continued metadata standardization. The use of structured export formats, standardized metadata fields, and openly documented workflows was therefore considered important to support long-term accessibility and future system transitions within institutional research data management infrastructures.

At the same time, the findings emphasize that successful digital transformation requires more than software deployment. It involves fostering a culture of transparency, promoting data literacy, and providing incentives for sustainable data practices. As observed in similar institutional initiatives [2,15], long-term acceptance depends on continuous engagement, recognition of user needs, and integration into existing scientific workflows.

Looking ahead, the University Medicine Halle plans to extend its ELN infrastructure toward interoperable research data management systems, thereby enabling seamless linkage between documentation, analysis, and archiving. This development will further support the creation of reproducible and verifiable research outputs, aligning local practices with international standards for data stewardship and open science.

In conclusion, the experience gained through this study provides a transferable, survey-informed onboarding framework for academic institutions aiming to implement electronic laboratory notebooks. By combining technical reliability, user-centered design, structured onboarding, and tool-supported migration, the implementation of eLabFTW at University Medicine Halle illustrates how institutional ELN adoption can be facilitated in a heterogeneous research environment.

## Supporting information

S1 Appendix Supplementary methods, survey instruments, and technical documentation. This appendix provides additional methodological and technical information referenced in the main manuscript, including the full survey item catalogue, complete descriptive response distributions, the onboarding framework and checklist, descriptions of the developed R Shiny tools for data migration and harmonization, migration workflow examples, supplementary framework material, and technical notes on analytics dashboards and metadata standardization strategies.
(PDF)
