# Supplementary material for: Digital transformation in laboratory documentation: Insights from an eLabFTW case study and a survey-informed onboarding framework
Source: PLoS One. 2026 Aug 20;21(8):e0355558. doi: 10.1371/journal.pone.0355558 (PMC13492770; doi:10.1371/journal.pone.0355558)
Supplement: Supplementary file 1 [file pone.0355558.s001.pdf]

S1 Appendix. Supplementary Information

Supporting Information for

Digital transformation in laboratory documentation: insights from an eLabFTW case study and a survey-informed onboarding framework

Christian Jäger et al.
Martin Luther University Halle-Wittenberg, 2026

This appendix provides additional methodological and technical information referenced throughout the main manuscript. It includes the complete survey item catalogue, full descriptive response distributions, onboarding framework documentation and checklists, descriptions of the developed R Shiny applications for data migration and harmonization, representative GUI screenshots, migration workflow examples, and supplementary technical notes on metadata standardization and FAIR-oriented documentation workflows.

Survey Items

S1 Table. Complete list of survey items and corresponding codes. The table reports the item code, thematic category, and a concise summary of each question as used for analysis.

Code	Category	Question Summary
G00Q01	Demographics	What is your position in your company/institute/department?
G00Q02	Demographics	In which field do you work?
G00Q03	Experience	How long have you been documenting your research data regardless of the format used, e.g., electronic or paper)?
G01Q01	Current Practice	What methods do you currently use to document your research? (Multiple selections possible)
G01Q02	Current Practice	(Perceived) Efficiency of current methods?
G01Q03	Current Practice	What challenges do you experience with your current documentation method? (Multiple selections possible)
G01Q04	Current Practice	How satisfied are you with your current method of documenting research results?
G01Q05	Current Practice	What technologies do you currently use to support your research documentation? (Multiple selections possible)
G01Q06	Improvement Needs	In which areas do you see the greatest need for improvement in your current documentation method? (Multiple selections possible)
G01Q07	Security	What security measures do you currently use to protect your research data? (Multiple selections possible)
G01Q08	Accessibility	How easy is it for you and your team to access the latest documentation?
G01Q09	Collaboration	How well does your current documentation method support collaboration and data exchange with colleagues?
G01Q10	Infrastructure	What electronic tools do you currently use to support your documentation? (Multiple selections possible)
G01Q11	Habits	How often do you update your research documentation?
G01Q12	ELN Adoption	What criteria are decisive for you in switching from a paper lab notebook to an ELN? (Multiple selections possible)
G01Q13	Paper Notebooks	What do you appreciate about using paper lab notebooks? (Multiple selections possible)
G01Q14	ELN Features	What features or characteristics would an ELN need to offer for you to consider switching? (Multiple selections possible)
G01Q15	Concerns	What concerns do you have about switching from a paper lab notebook to an ELN? (Multiple selections possible)
G01Q16	Support	What type of support or training would you require to successfully transition to an ELN? (Multiple selections possible)
G02Q01	Reason	For what reason have you not used electronic documentation of research data so far?
G02Q02	Motivation	Why are you planning or required to start electronic documentation of research data soon? (Multiple answers possible)
G02Q03	Data Types	Which types of research data will you likely document electronically? (Multiple answers possible)
G02Q04	Concerns	What are your main concerns regarding the electronic documentation of research data? (Multiple answers possible)
G02Q05	Support Needed	What support or resources would help you get started with electronic documentation? (Multiple answers possible)
G02Q06	Key Features	Which features do you consider important when starting electronic documentation? (Multiple answers possible)
G02Q07	Urgency	How soon do you want or need to begin electronic documentation?
G02Q08	Main Benefit	What do you believe will be the greatest benefit of documenting your research data electronically? (Multiple answers possible)
G00Q04	Comment	further comments

Supplementary Results: Detailed Descriptive Statistics

S2 Table. G00Q01 - Full list of role options provided in the survey (single choice).

Role
Professor
Junior Professor
Privatdozent (Private Lecturer)
Doctoral Student (PhD Student)
Postdoctoral Researcher (Postdoc)
Habilitation Candidate
Scientific Staff Member
Visiting Researcher
Laboratory Head
Biological Technical Assistant (BTA)
Medical Technical Assistant (MTA)
Chemical Technical Assistant (CTA)
Pharmaceutical Technical Assistant (PTA)
Laboratory Engineer
Research Coordinator
Laboratory Technician
Biostatistician
Animal Caretaker (Laboratory Animal Technician)
Research Officer
Project Manager
Research Administrator
Study Coordinator
Grant Writer
Senior Physician
Resident Physician
Specialist Physician
Clinical Researcher
Student
Other

G00Q02 – Disciplinary affiliation

Individual free-text responses are not reproduced in full due to the potential risk of participant re-identification. Aggregated disciplinary categories are summarized in the main manuscript.

S3 Table. G00Q03 - Experience with research data documentation.

Experience Level	# Responses	Percentage
More than 5 years	43	78.18%
3–5 years	3	5.45%
1–3 years	5	9.09%
Less than 1 year	1	1.82%
No experience	3	5.45%

S4 Table. G01Q02 - (Perceived) efficiency of current documentation methods.

Efficiency Level	# Responses	Percentage
Very Efficient	2	3.85%
Efficient	24	46.15%
Neutral	19	36.54%
Less Efficient	5	9.62%
Inefficient	2	3.85%

S5 Table. G01Q03 - Challenges with current documentation methods.

Challenge	# Responses	Percentage
Time Consumption	35	67.31%
Difficulties in Data Organization	33	63.46%
Problems in Finding Information	33	63.46%
Lack of Data Security	13	25.00%
Other	8	15.38%

S6 Table. G01Q04 - Satisfaction with current documentation methods.

Satisfaction Level	# Responses	Percentage
Very Satisfied	1	1.92%
Satisfied	19	36.54%
Neutral	22	42.31%
Dissatisfied	9	17.31%
Very Dissatisfied	1	1.92%

S7 Table. G01Q05 - Technologies used to support documentation.

Technology	# Responses	Percentage
Desktop Computers	43	82.69%
Paper-Based Laboratory Notebooks	40	76.92%
Laptops	22	42.31%
Tablets/Smartphones	3	5.77%

S8 Table. G01Q07 - Security measures in place.

Measure	# Responses	Percentage
Regular Backups	30	57.69%
Password Protection	28	53.85%
Physical Security (e.g., lockable cabinets)	16	30.77%
No Special Measures	12	23.08%
Other	2	3.85%

S9 Table. G01Q08 - Ease of access to the latest documentation.

Ease of Access	# Responses	Percentage
Very Easy	2	3.85%
Easy	25	48.08%
Neutral	20	38.46%
Difficult	5	9.62%
Very Difficult	0	0.00%

S10 Table. G01Q09 - Support for collaboration and data exchange.

Support Level	# Responses	Percentage
Very Good	0	0.00%
Good	11	21.15%
Neutral	19	36.54%
Poor	18	34.62%
Very Poor	4	7.96%

S11 Table. G01Q10 - Utilized electronic tools.

Tool	# Responses	Percentage
Network Drives	48	92.31%
Removable Media (e.g., USB Sticks)	32	61.54%
Cloud Storage	18	34.62%
Databases	10	19.23%
None	2	3.85%
Other	4	7.69%

S12 Table. G01Q11 - Frequency of documentation updates.

Frequency	# Responses	Percentage
Daily	23	44.23%
Weekly	11	21.15%
Monthly	8	15.38%
Rarely	8	15.38%
Never	2	3.85%

S13 Table. G01Q13 - Advantages of paper-based lab notebooks.

Advantage	# Responses	Percentage
Flexibility in Writing and Drawing	38	73.08%
Ease of Use	37	71.15%
No Technical Issues	30	57.69%
Habit	28	53.85%
Other	8	15.38%

S14 Table. G01Q14 - Desired ELN features.

Feature	# Responses	Percentage
Ease of Use	49	94.23%
Customizable Templates	41	78.85%
Automatic Data Backup	38	73.08%
Offline Access	31	59.62%
Collaboration Capabilities	26	50.00%
Other	4	7.69%

S15 Table. G01Q15 - Concerns about switching to the ELN.

Concern	# Responses	Percentage
Insufficient User-Friendliness	36	69.23%
Familiarization Period	31	59.62%
Technical Difficulties	29	55.77%
Lack of Integration with Lab Processes	24	46.15%
Data Loss	16	30.77%
Costs	7	13.46%
Data Privacy Concerns	3	5.77%
Other	8	15.38%

Onboarding Framework Overview

This section provides a structured overview of the onboarding framework developed to support the introduction of eLabFTW at University Medicine Halle. It expands upon the methodological details discussed in the main text and outlines the organizational measures, role definitions, and integration steps implemented during the rollout.

Onboarding Checklist

S16 Table. Onboarding checklist used for introducing eLabFTW to new research teams.

Section	Question / Task	Details and Notes	Comments / Log
**A** Preparation	What types of documentation are needed?	Experiments, samples, device maintenance, analysis reports.	
	Are there existing paper templates?	Should they be digitized and implemented as eLabFTW templates?	
	What does the current paper-based workflow look like?	Individual or centralized documentation; include failed experiments?	
	Are all users registered in eLabFTW?	Ensure access levels based on roles.	
	Are admin rights assigned?	Define admins for template and permissions management.	
	Are example templates prepared?	Example: sample management, device logs.	
	Naming conventions and folder translation?	Example project name: 2024-ProteinDesign-Project. Use of tags and project structures.	
**B** Goals and Expectations	What improvements are expected?	More efficient documentation, faster retrieval, automation.	
	What are the system requirements?	Ease of use, platform independence, export options.	
	What are user concerns?	Usability, acceptance among team members.	
	Who are the primary users?	Lab staff, project leads, analysts.	
	Are there secondary stakeholders?	Collaborating groups, industry partners.	
	How will goal achievement be measured?	Review after 6–12 months; metrics: time and entry quality.	
	What if goals are not met?	Plan for system adjustments or additional training.	
**C** Roles and Responsibilities	Who are the administrators?	Manage templates, categories, and access rights.	
	Who are the power users?	Provide team-level support and serve as local contacts.	
**D** Template Development	Which templates are needed?	For example: experiments, storage, device maintenance.	
	Are headings and fields standardized?	Fields: project title, experiment ID, date range.	
	Has a test run been conducted?	Create sample entries and collect feedback.	
**E** Naming Conventions	How should entries be named?		
	Experiments:	[Project]_[Date]_[Description]_[RunNr]	
	Samples:	[Type]_[ID]_[Storage]	
	Maintenance:	[Device]_[Date]_[Type]	
	Reports:	[Project]_[Method]_[Version]	
	Are abbreviations standardized?	Use documented acronyms (PCR, WB, etc.).	
	Numbering of repeats?	Example: Test1, Test2.	
**F** Workflow Integration	Are documentation standards defined?	Structure: Introduction, Methods, Results.	

(continued from previous page)

Section	Question / Task	Details and Notes	Comments / Log
	Are naming rules accessible?	Stored in Confluence or eLabFTW.	
	When and how are templates used?	During experiment or by key users.	
	Who is responsible for QA?	Define responsible roles (for example lab manager).	
	How does QA work?	Create → Review → Sign → Archive.	
**G** Automation and Interfaces	API-ready devices present?	For example balances, HPLC, microscopes.	
	How will data transfer work?	Evaluate eLabFTW API and device plugin options.	
**H** Collaboration and Access Control	With whom is collaboration planned?	Define access levels by project or team.	
	How is access managed?	Use of role-based permissions and groups.	
**I** Feedback and Iteration	Is feedback collected?	Are templates and naming conventions practical?	
	Are changes planned?	Adjust based on experience and feedback.	
**J** Next Steps	What tasks remain?	Finalize templates, create test entries, gather feedback.	
	When is follow-up planned?	Review in 4–6 weeks.	

R Shiny Applications for Data Migration and Harmonization

Two R Shiny applications were developed to support the structured migration and integration of legacy laboratory data into eLabFTW. They enable standardized data import, metadata generation, and harmonization across heterogeneous sources. Detailed user documentation and source code are available in open repositories.

S17 Table. Comparison of developed R Shiny applications.

Feature	Excel-to-CSV Converter	CSV/SQL Merger
Input formats	Excel (.xlsx)	CSV, TXT, optional SQL dump
Output formats	CSV with JSON metadata	CSV or Excel
Purpose	Metadata generation and standardization	Data consolidation and harmonization
Mapping logic	Field mapping via user interface	ID-based joins (inner, outer, left, right)
Intended users	General lab staff	Data managers or advanced users
Integration target	eLabFTW import interface	Preprocessing for eLabFTW

GUI Screenshots

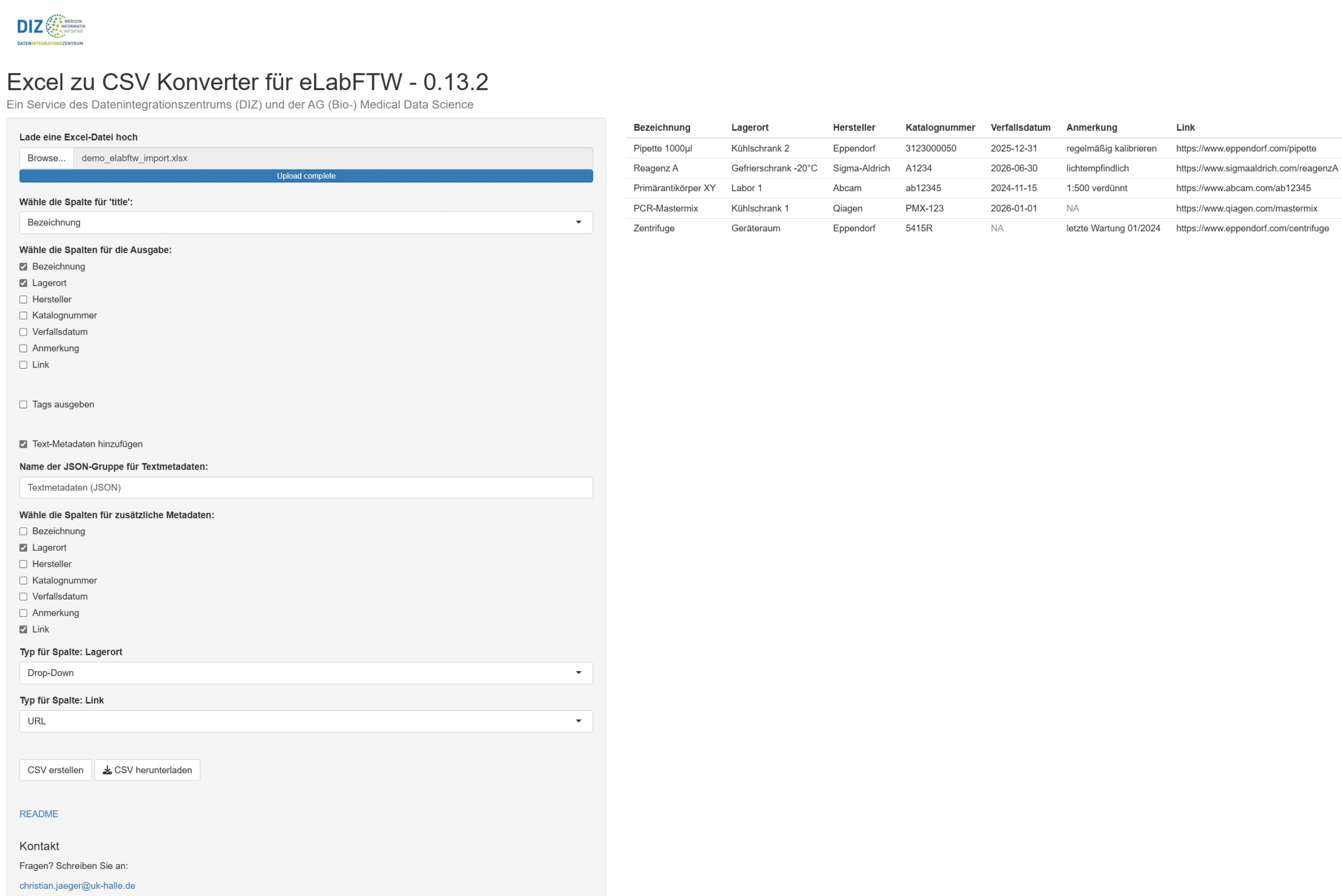

S1 Fig. Excel-to-CSV Converter user interface. Interactive mapping of spreadsheet columns and metadata fields for structured import into eLabFTW.

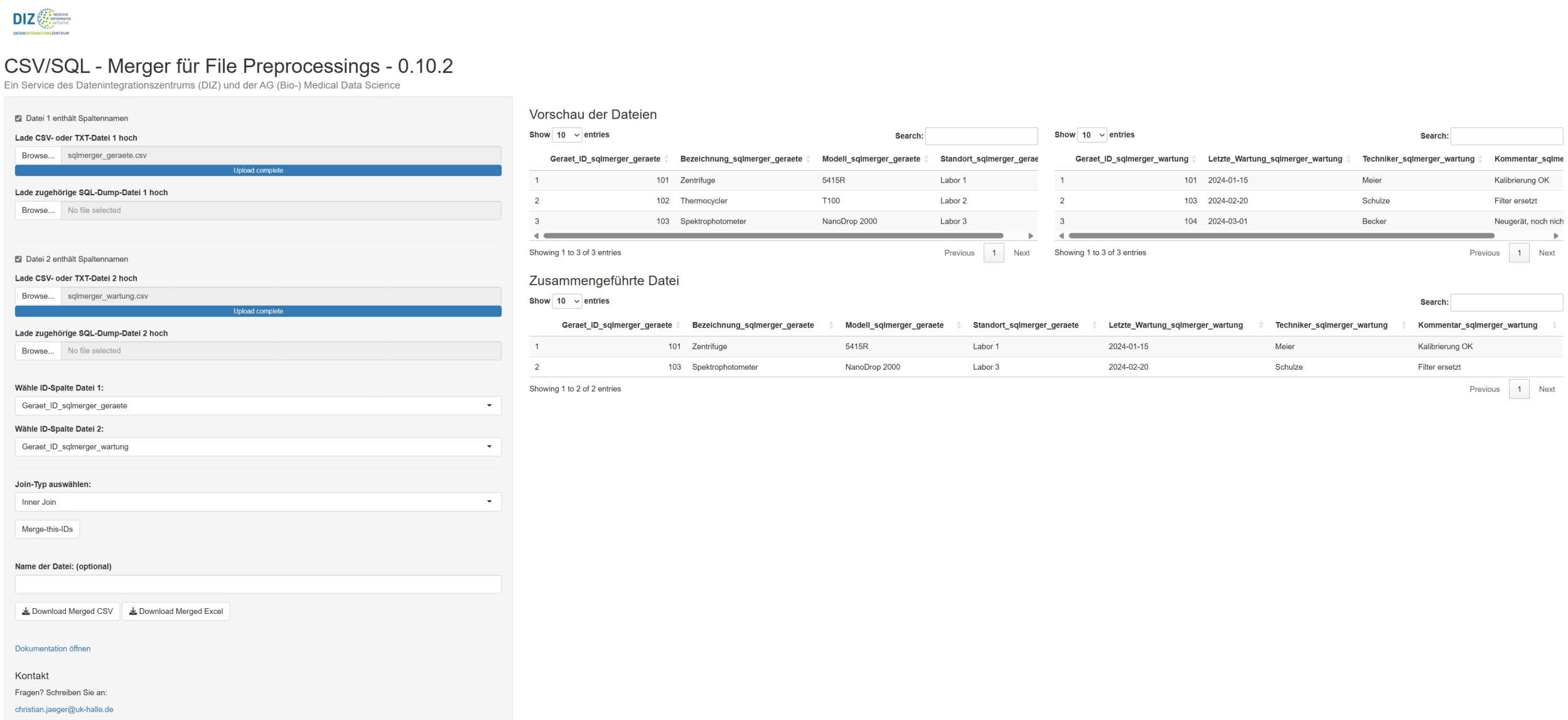

S2 Fig. CSV/SQL Merger user interface. Preview window for merged datasets and join-type configuration.

Apache Superset Dashboards for eLabFTW Analytics

Apache Superset was deployed as an additional visualization layer to enable interactive dashboards and automated reporting based on the complete eLabFTW data structure. For security and performance reasons, Superset does not query the production database directly. Instead, it accesses a regularly synchronized, read-only clone of the eLabFTW database, ensuring data integrity, preventing interference with routine ELN operations, and enabling controlled analytics workflows.

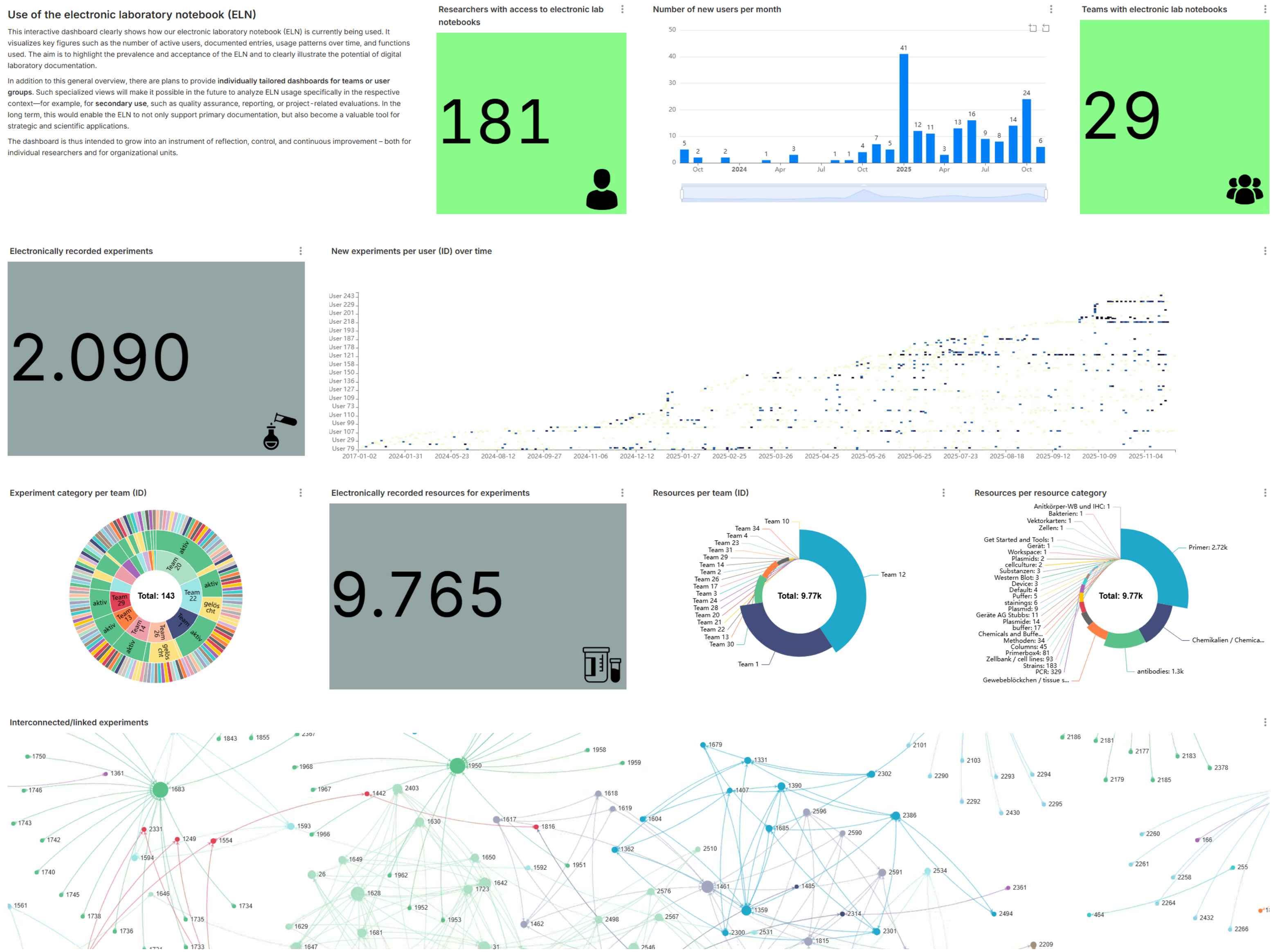

S3 Fig. Example Apache Superset dashboard for eLabFTW analytics. Dashboard visualizing usage patterns and metadata completeness across a cloned eLabFTW database.
